# Population Pharmacokinetic and Pharmacokinetic/Pharmacodynamic Analyses of Cefiderocol, a Parenteral Siderophore Cephalosporin, in Patients with Pneumonia, Bloodstream Infection/Sepsis, or Complicated Urinary Tract Infection

**DOI:** 10.1128/AAC.01437-20

**Published:** 2021-02-17

**Authors:** Nao Kawaguchi, Takayuki Katsube, Roger Echols, Toshihiro Wajima

**Affiliations:** a Clinical Pharmacology & Pharmacokinetics, Shionogi & Co., Ltd., Osaka, Japan; b Infectious Disease Drug Development Consulting, LLC, Easton, Connecticut, USA

**Keywords:** augmented renal function, bloodstream infections, cefiderocol, cephalosporin, complicated urinary tract infection, pharmacodynamics, pneumonia, population pharmacokinetics, ventilation

## Abstract

Cefiderocol is a novel siderophore cephalosporin with antibacterial activity against Gram-negative bacteria, including carbapenem-resistant strains. The standard dosing regimen of cefiderocol is 2 g administered every 8 hours over 3 hours infusion in patients with creatinine clearance (CrCL) of 60 to 119 ml/min, and it is adjusted for patients with <60 ml/min or ≥120 ml/min CrCL. A population pharmacokinetic (PK) model was constructed using 3,427 plasma concentrations from 91 uninfected subjects and 425 infected patients with pneumonia, bloodstream infection/sepsis (BSI/sepsis), and complicated urinary tract infection (cUTI). Plasma cefiderocol concentrations were adequately described by the population PK model, and CrCL was the most significant covariate. No other factors, including infection sites and mechanical ventilation, were clinically relevant, although the effect of infection sites was identified as a statistically significant covariate in the population PK analysis. No clear pharmacokinetic/pharmacodynamic relationship was found for any of the microbiological outcome, clinical outcome, or vital status. This is because the estimated percentage of time for which free plasma concentrations exceed the minimum inhibitory concentration (MIC) over dosing interval (%*f*T_>MIC_) was 100% in most of the enrolled patients. The probability of target attainment (PTA) for 100% *f*T_>MIC_ was >90% against MICs of ≤4 μg/ml for all infection sites and renal function groups except for BSI/sepsis patients with normal renal function (85%). These study results support adequate plasma exposure can be achieved at the cefiderocol recommended dosing regimen for the infected patients, including the patients with augmented renal function, ventilation, and/or severe illness.

## Abstract (precis)

Cefiderocol is a novel siderophore cephalosporin with antibacterial activity against Gram-negative bacteria, including carbapenem-resistant strains. The standard dosing regimen of cefiderocol is 2 g administered every 8 hours over 3 hours infusion in patients with creatinine clearance (CrCL) of 60 to 119 ml/min, and it is adjusted for patients with <60 ml/min or ≥120 ml/min CrCL.

## TEXT

Cefiderocol is a novel parenteral siderophore cephalosporin discovered and developed by Shionogi & Co., Ltd. Cefiderocol exhibits antibacterial activity against a wide range of carbapenem-susceptible and carbapenem-resistant Gram-negative bacteria, including *Enterobacterales*, Acinetobacter baumannii, Pseudomonas aeruginosa, and Stenotrophomonas maltophilia (1–5). The activity is attributed to its unique structural features with a catechol substituent that chelates ferric iron and utilizes the bacterial active iron transport system to penetrate the outer membrane of Gram-negative bacteria.

Cefiderocol was approved in the United States for the treatment of hospital-acquired bacterial pneumonia, ventilator-associated bacterial pneumonia, and complicated urinary tract infections (cUTIs), including pyelonephritis, caused by susceptible Gram-negative microorganisms in patients ≥18 years of age (6). Cefiderocol was also approved by the European Medicines Agency in April 2020 for the treatment of infections due to aerobic Gram-negative organisms in adults with limited treatment options (7). The approved dosing regimens are based on a creatinine clearance estimated by the Cockcroft-Gault equation (CrCL) (8) since cefiderocol is primarily excreted unchanged via the kidneys and the total clearance (CL) is dependent on renal function (9–12). The standard dosing regimen is 2 g administered every 8 hours (q8h) over 3 hours infusion in adults with CrCL of 60 to 119 ml/min, and it is adjusted for patients with CrCL less than 60 ml/min or for patients with CrCL 120 ml/min or greater (6, 7).

The pharmacokinetic/pharmacodynamic (PK/PD) index of cefiderocol is the percentage of time for which free drug concentrations in plasma exceed the minimum inhibitory concentration (MIC) over dosing interval (%*f*T_>MIC_) (5), as reported in other cephalosporins (13–15). The mean %*f*T_>MIC_ values required for a 1-log_10_ reduction against *Enterobacterales* and P. aeruginosa in the thigh infection model were 73% and 77%, respectively. The mean %*f*T_>MIC_ values against *Enterobacterales*, P. aeruginosa, A. baumannii, and S. maltophilia in the lung infection model were 64%, 70%, 88%, and 54%, respectively. The estimated value was higher for carbapenem-resistant strains (85%) than for carbapenem-susceptible strains (61%) (5). The *in vitro* plasma protein binding, primarily to albumin, of cefiderocol in human is 57.8% (16).

Previously published population PK analyses of cefiderocol were performed based on concentration data in healthy subjects, subjects with various degrees of renal function, and patients with cUTI or acute uncomplicated pyelonephritis (AUP) enrolled in the APEKS-cUTI study (https://www.clinicaltrials.gov identifier NCT02321800) (10, 12, 17). Recently, two phase 3 clinical trials, the CREDIBLE-CR study (https://www.clinicaltrials.gov identifier NCT02714595) (18) and the APEKS-NP study (https://www.clinicaltrials.gov identifier NCT03032380) (19), were completed. In the CREDIBLE-CR study, the microbiological and clinical outcomes were comparable between cefiderocol and best available therapy groups in patients with pneumonia, bloodstream infection/sepsis (BSI/sepsis), or cUTI caused by carbapenem-susceptible or carbapenem-resistant Gram-negative pathogens. In the APEKS-NP study, noninferiority of cefiderocol to meropenem following 2 g every 8 hours (q8h) over 3 hours infusion was demonstrated in patients with pneumonia caused by Gram-negative pathogens. A summary of study designs in each clinical study is shown in Table S1 in the supplemental material.

The objective of this study was to build an updated population PK model using plasma cefiderocol concentration data from patients with pneumonia, BSI/sepsis, and cUTI/AUP caused by Gram-negative pathogens; subjects with various degrees of renal function; and healthy subjects. A PK/PD analysis was also conducted to assess the relationships of %*f*T_>MIC_ with microbiological outcome, clinical outcome, and vital status in the patients from the phase 3 studies. Furthermore, the probability of target attainment (PTA) for target %*f*T_>MIC_ was calculated to assess the cefiderocol recommended dosing regimen adjusted based on renal function.

## RESULTS

A population PK model was developed using data of 3,427 plasma cefiderocol concentrations from 516 subjects (Fig. S1 in the supplemental material). A summary of background characteristics for the analysis population is shown in Table 1.

**TABLE 1:** Background characteristics for population pharmacokinetic analysis population^*a*^

Characteristic	Data for:			
	Phase 1 studies (*n = *91)	Phase 2 APEKS-cUTI study (*n = *238)	Phase 3 APEKS-NP study (*n = *115)	Phase 3 CREDIBLE-CR study (*n = *72)
Body weight (median [range] [kg])	68.4 (45.1–124.1)	76.4 (46.3–138.0)	72.0 (28.9–130.0)	68.4 (25.0–156.0)
Age (median [range] [yrs])	36.0 (20–74)	65.0 (18–93)	68.0 (18–91)	67.5 (21–92)
eGFRadj (median [range] [ml/min/1.73 m^2^])	99.0 (4–146)	72.0 (14–142)	72.0 (6–225)	82.0 (15–507)
eGFRabs (median [range] [ml/min])	99.0 (5–144)	78.0 (16–148)	76.0 (4–283)	81.0 (13–533)^*f*^
CrCL (median [range] [ml/min])	121.0 (7–185)	83.0 (25–186)	69.0 (5–306)	73.0 (10–540)
Albumin (median [range] [g/dl])	4.2 (3.1–4.8)	4.2 (2.5–5.3)	3.0 (1.2–4.5)^*d*^	2.7 (1.6–4.8)^*g*^
Aspartate aminotransferase (median [range] [U/liter])	18.0 (10–45)	18.0 (6–101)	27.6 (3–139)^*e*^	36.0 (10–367)^*f*^
Alanine aminotransferase (median [range] [U/liter])	18.0 (5–51)	15.0 (4–111)	26.4 (4–116)^*e*^	26.5 (6–153)
Total bilirubin (median [range] [mg/dl])	0.78 (0.20–2.00)	0.53 (0.19–2.88)	0.64 (0.10–2.26)^*e*^	0.56 (0.15–15.20)^*h*^
Sex (no. male, no. female)^*b*^	75 (82.4), 16 (17.6)	108 (45.4), 130 (54.6)	78 (67.8), 37 (32.2)	48 (66.7), 24 (33.3)
Race^*b*^				
White, non-White	23 (25.3), 68 (74.7)	230 (96.6), 8 (3.4)	78 (67.8), 37 (32.2)	42 (58.3), 30 (41.7)
Asian, White, Black, Native, other	49 (53.9), 23 (25.3), 17 (18.7), 1 (1.1), 1 (1.1)	7 (2.9), 230 (96.6), 0 (0.0), 0 (0.0), 1 (0.4)	36 (31.3), 78 (67.8), 0 (0.0), 0 (0.0), 1 (0.9)	22 (30.6), 42 (58.3), 0 (0.0), 0 (0.0), 8 (11.1)
Infection (pneumonia, BSI/sepsis, cUTI, no infection)^*b*^	0 (0.0), 0 (0.0), 0 (0.0), 91 (100.0)	0 (0.0), 0 (0.0), 238^*c*^ (100.0), 0 (0.0)	115 (100.0), 0 (0.0), 0 (0.0), 0 (0.0)	31 (43.1), 20 (27.8), 21 (29.2), 0 (0.0)

a CrCL, creatinine clearance calculated by Cockcroft-Gault equation; eGFRabs, absolute estimated glomerular filtration rate; eGFRadj, body surface area-adjusted estimated glomerular filtration rate; Black, Black or African American; Native, native American or Alaska native.

b Shown is the number (percentage) for each value.

c Patients with cUTI or AUP.

d *n* = 112.

e *n* = 113.

f *n* = 71.

g *n* = 69.

h *n* = 68.

**FIG 1:**
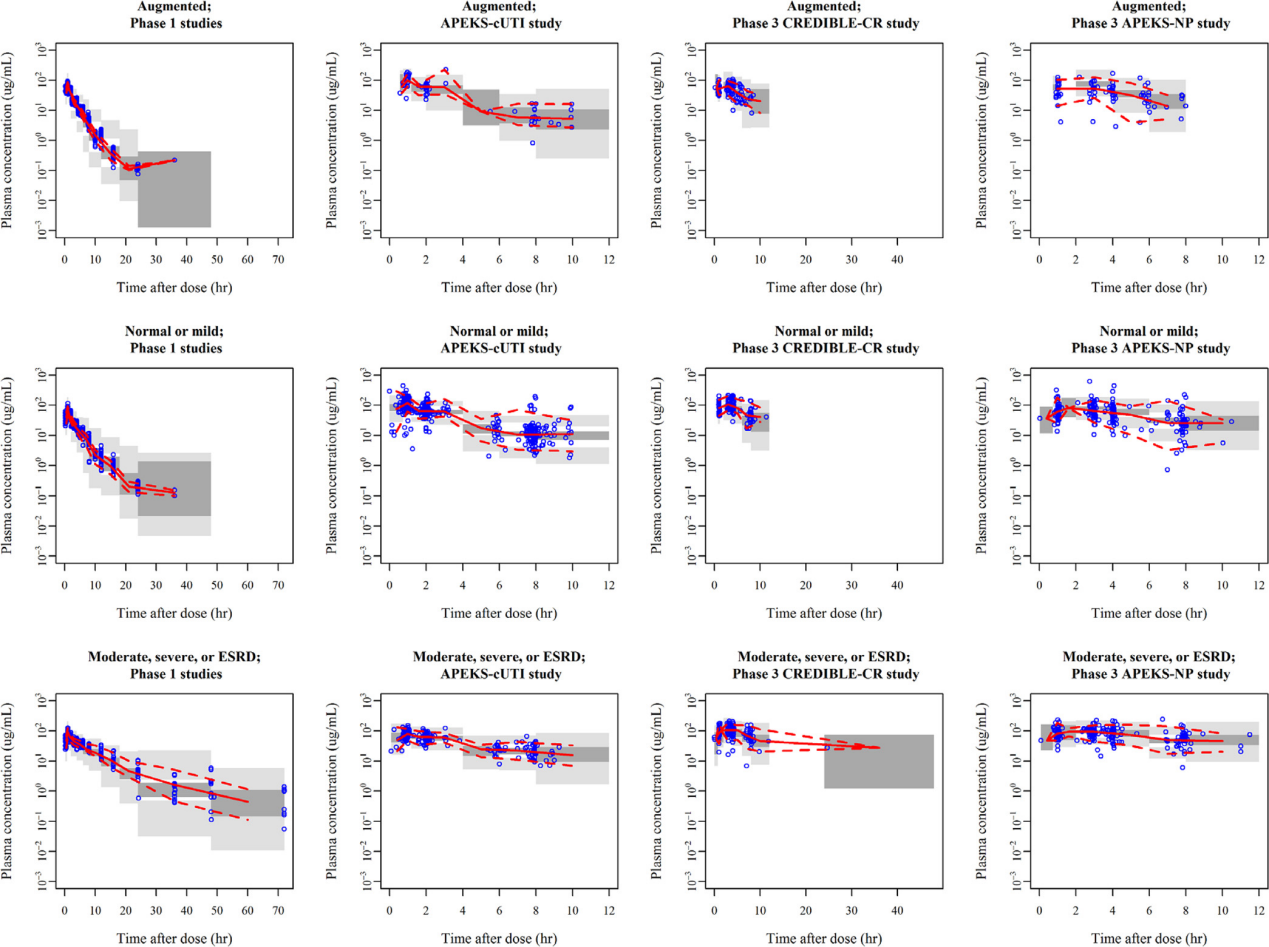
Prediction-corrected visual predictive check for final model by study and renal function group. Results for 500 simulations. Renal function groups defined by CrCL were as follows: augmented, ≥120 ml/min; normal or mild, 60 to <120 ml/min; moderate, severe, or end-stage renal disease (ESRD), 5 to <60 ml/min. Semilog scale. Solid line, observed median; dashed line, observed 2.5th and 97.5th percentiles; dark-gray shaded area, model-predicted 95% confidence interval of median; gray shaded area, model predicted 95% confidence intervals of 2.5th and 97.5th percentiles.

Plasma cefiderocol concentrations were adequately described by a 3-compartment model with a proportional error model for the intraindividual variability. The developed final model contained the effects of CrCL and infection sites (pneumonia [CREDIBLE-CR and APEKS-NP studies], BSI/sepsis, cUTI [CREDIBLE-CR study], or cUTI/AUP [APEKS-cUTI study]) on CL, body weight on the volume of distribution in the central and peripheral compartments (*V*_1_ and *V*_2_, respectively), albumin concentration (ALB), and any of the infection sites on *V*_1_. The model code and parameter estimates are shown in Table S2 and Table 2, respectively. Goodness-of-fit (GOF) plots for the final model demonstrated good fitting to the data without any bias (Fig. S2). Prediction-corrected visual predictive check (pcVPC) indicated that the model well captured the central tendency and variability of the observed data (Fig. 1). The parameter estimates for the final model were comparable to the median of bootstrap estimates (Table 2), suggesting the robustness of the final model.

**TABLE 2:** Population pharmacokinetic parameter estimates for final model^*a*^

Pharmacokinetic parameter	Final model^*b*^		Bootstrap estimates	
	Estimate	%RSE	Median	95% CI
CL (liter/h)	4.04	1.8	4.04	3.89 to 4.20
*V*_1_ (liter)	7.78	5.2	7.93	7.07 to 8.85
*Q*_2_ (liter/h)	6.19	5.7	5.97	4.57 to 7.24
*V*_2_ (liter)	5.77	3.2	5.68	5.02 to 6.15
*Q*_3_ (liter/h)	0.127	14.1	0.119	0.0792 to 0.228
*V*_3_ (liter)	0.798	6.4	0.772	0.621 to 1.09
Effect of CrCL on CL (CrCL cutoff value of 150 ml/min)	0.682	4.0	0.681	0.626 to 0.735
Effect of body weight on *V*_1_ and *V*_2_	0.580	12.2	0.571	0.433 to 0.725
Effect of infection with pneumonia on CL	0.981	4.1	0.978	0.893 to 1.07
Effect of infection with BSI/sepsis on CL	1.08	10.4	1.07	0.894 to 1.37
Effect of infection with cUTI in CREDIBLE-CR study on CL	0.872	6.4	0.869	0.769 to 1.01
Effect of infection with cUTI/AUP in APEKS-cUTI study on CL	1.27	3.1	1.27	1.20 to 1.35
Effect of albumin concentration on *V*_1_	−0.617	10.9	−0.624	−0.985 to −0.244
Effect of infection on *V*_1_	1.39	6.7	1.36	1.22 to 1.54
Interindividual variability (CV% [sh_ηp])				
CL	37.5 (3.6)	10.4	37.0	32.9 to 40.7
*V*_1_	56.9 (13.6)	19.8	57.9	45.3 to 71.0
*V*_2_	33.6 (18.2)	35.0	35.5	19.7 to 50.2
Covariance between CL and *V*_1_	0.0886^*c*^	29.1	0.0807	0.0338 to 0.146
Covariance between CL and *V*_2_	0.0792^*d*^	33.2	0.0767	0.0187 to 0.140
Covariance between *V*_1_ and *V*_2_	0.150^*e*^	27.3	0.115	−0.0930 to 0.218
Intraindividual variability (CV% [sh_ε])				
Proportional residual error	20.5 (13.2)	5.1	20.3	18.5 to 22.5

a CV, coefficient of variation; sh_ηp, shrinkage in the standard deviation of interindividual variability parameters η; sh_ε, shrinkage in the standard deviation of intraindividual variability parameters ε; %RSE, relative standard error in percent; *R*, coefficient of correlation; CI, confidence interval.

b For CrCL < 150 ml/min, CL = 4.04 × (CrCL/83.0)^0.682^ × (0.981 for patients with pneumonia) × (1.08 for patients with BSI/sepsis) × (0.872 for patients with cUTI in CREDIBLE-CR study) × (1.27 for patients with cUTI/AUP in APEKS-cUTI study). For CrCL > 150 ml/min, CL = 4.04 × (150/83.0)^0.682^ × (0.981 for patients with pneumonia) × (1.08 for patients with BSI/sepsis) × (0.872 for patients with cUTI in CREDIBLE-CR study) × (1.27 for patients with cUTI/AUP in APEKS-cUTI study). *V*_1_ = 7.78 × (body weight/72.6)^0.580^ × (albumin/3.9)^−0.617^ × (1.39 for patients with infection). *V*_2_ = 5.77 × (body weight/72.6)^0.580^.

c *R* = 0.415.

d *R* = 0.629.

e *R* = 0.784.

CrCL was the most significant covariate on cefiderocol PK, as expected from the previous analysis (12). The CL of cefiderocol was assumed to increase following the power model for up to 150 ml/min CrCL with constant CL for ≥150 ml/min CrCL. The CrCL cutoff value of 150 ml/min was selected based on visual inspection of the relationship between CL and CrCL (Fig. S3). Then, the appropriateness of the selected value (150 ml/min) was confirmed by testing three cutoff values of 120, 150, and 180 ml/min and comparing their model fitting based on the values of objective function (OBJ). A negative correlation between ALB and *V*_1_ was observed. The CL in patients with pneumonia, BSI/sepsis, and cUTI (CREDIBLE-CR study) was comparable to that in subjects without infection. In contrast, the CL in patients with cUTI/AUP in the APEKS-cUTI study was 27% higher than that in subjects without infection, which was consistent with the previous analysis (12). The *V*_1_ in infected patients was suggested to be 39% higher than that in subjects without infection.

**FIG 2:**
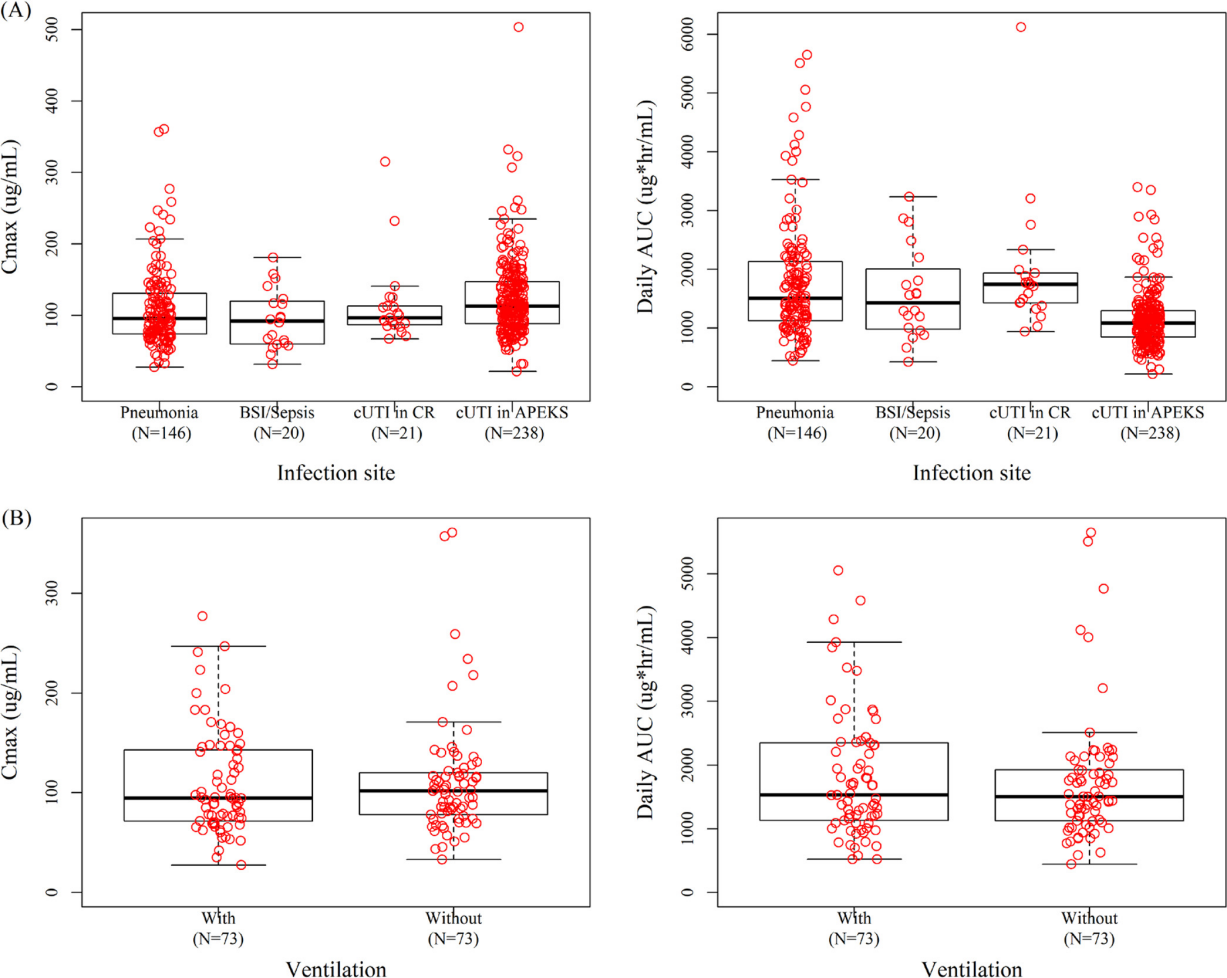
Box plots for estimated *C*_max_ and daily AUC by infection site (A) and ventilation status in pneumonia patients (B). cUTI in CR, cUTI in CREDIBLE-CR study; cUTI in APEKS, cUTI/AUP in APEKS-cUTI study. Red circle, *post hoc* estimates of parameters for individual patients. Horizontal black center line represents median, with the top and the base of the boxes representing first and third quartiles (interquartile range [IQR]); whiskers represent the most extreme data within 1.5 times the IQR.

The maximum concentration (*C*_max_) and daily area under the concentration-time curve (AUC) calculated using empirical Bayesian estimation overlapped among infection sites (Fig. 2A). For pneumonia patients, the estimated *C*_max_ and AUC were similar between the patients with and without mechanical ventilation, as shown in Fig. 2B.

**FIG 3:**
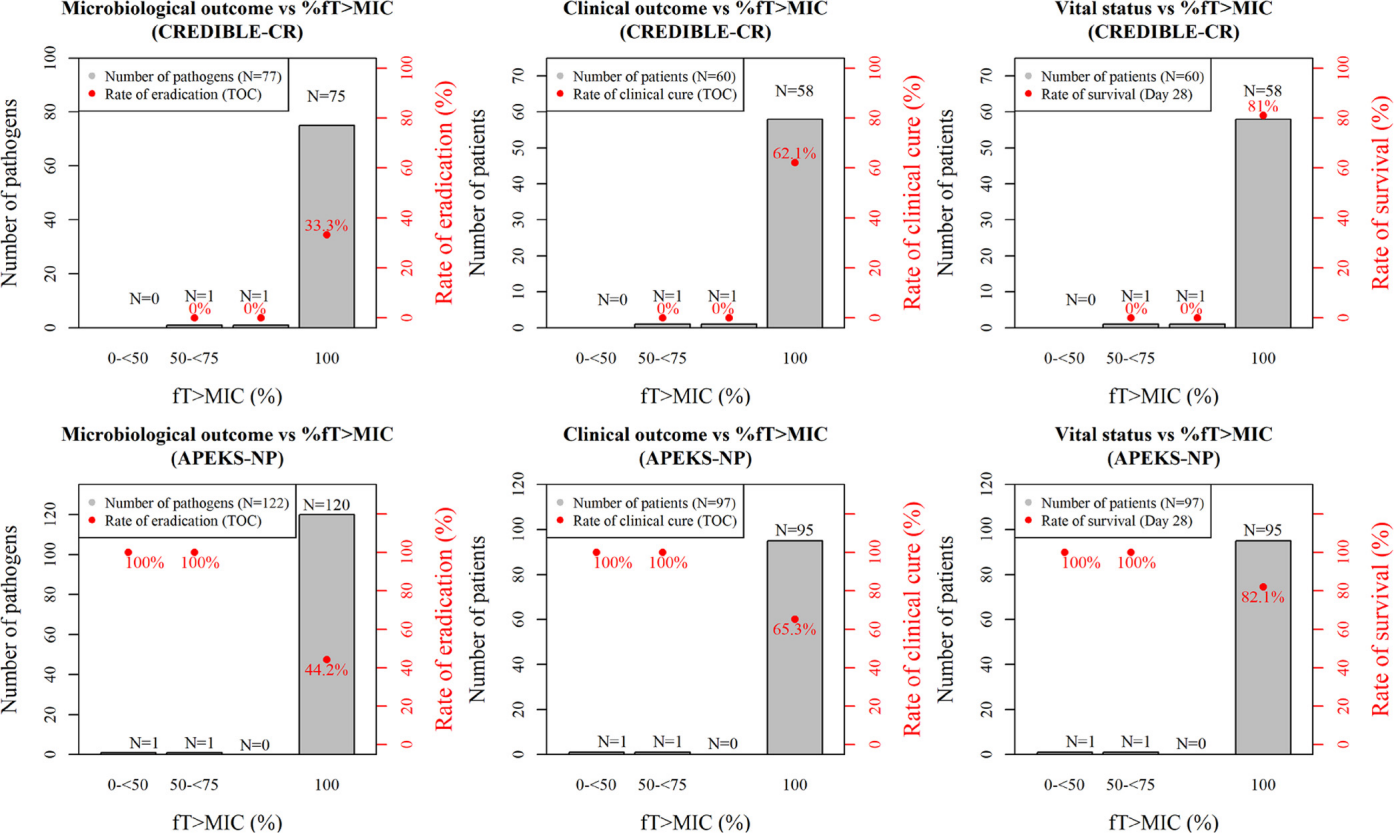
Relationships of %*f*T_>MIC_ with microbiological outcome, clinical outcome, or vital status in CREDIBLE-CR and APEKS-NP studies.

A PK/PD analysis was conducted using the data from 60 patients in the CREDIBLE-CR study and 97 patients in the APEKS-NP study. Total numbers of isolated pathogens at baseline in the CREDIBLE-CR and APEKS-NP studies were 77 and 122, respectively, and approximately 30% of the patients were infected by more than one Gram-negative pathogen. The range (median) of MIC of the isolated Gram-negative pathogens was ≤0.03 to 64 μg/ml (0.25 μg/ml) in both studies (Table S3). The %*f*T_>MIC_ was 100% in 97% of the patients in both studies. No clear PK/PD relationship was found for any of the outcomes or vital status. This is because the %*f*T_>MIC_ was 100% in most of the patients in the phase 3 studies (Fig. 3).

**FIG 4:**
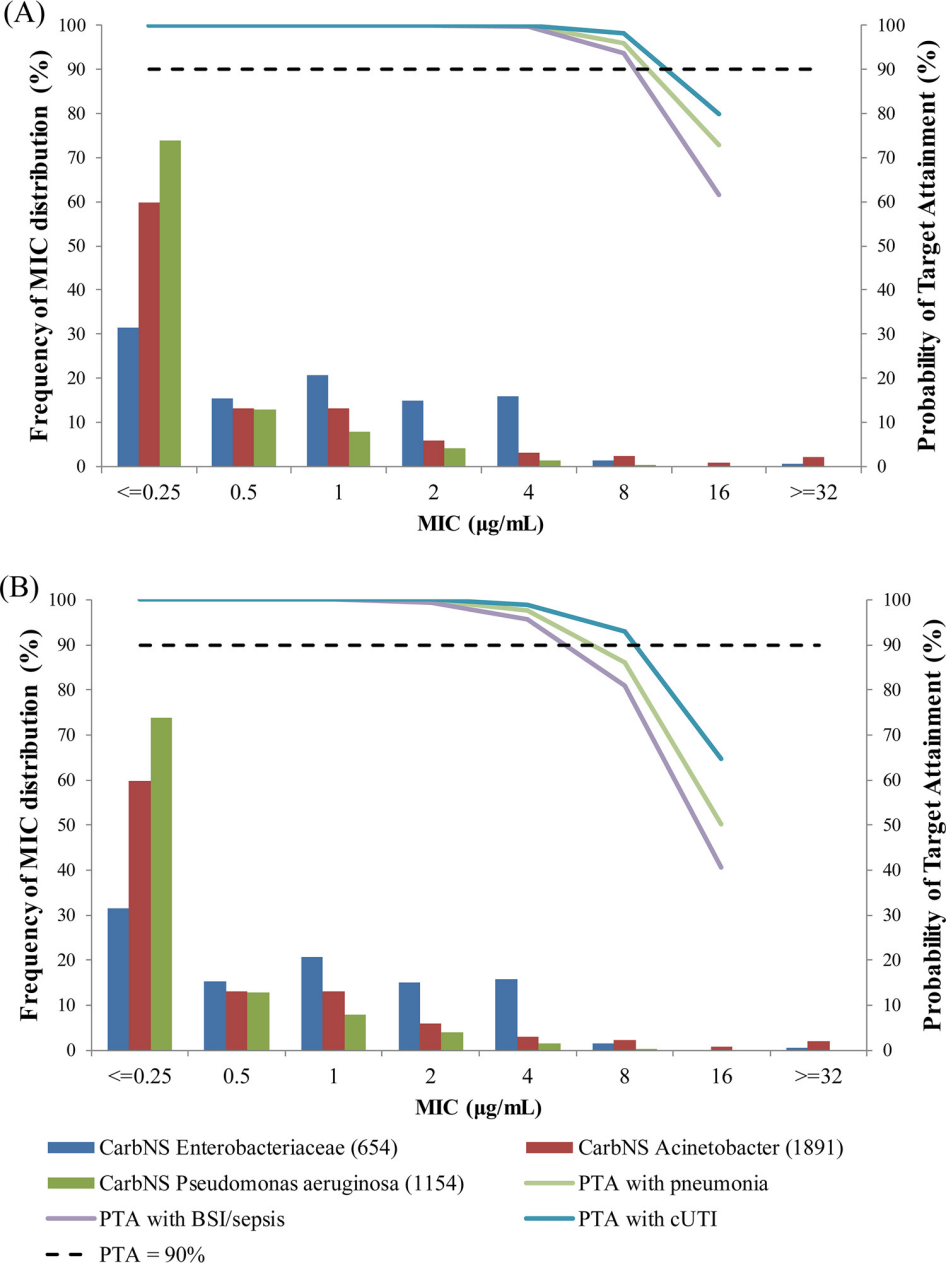
Integrated probability of target attainment for 75% *f*T_>MIC_ (A) and 100% *f*T_>MIC_ (B) calculated by weighting for distribution of creatinine clearance in phase 3 studies.

The PTAs for 75% *f*T_>MIC_ and 100% *f*T_>MIC_ were calculated in the simulated patients with different infection sites and renal function groups. The PTA for 75% *f*T_>MIC_ was >95% against MICs ≤4 μg/ml regardless of infection site or renal function group (Tables 3 and 4). The PTA even for 100% *f*T_>MIC_ was >90% against MICs ≤4 μg/ml for all of the infection sites and renal function groups except for the normal renal function in BSI/sepsis patients (85%). The PTA integrated with all renal function groups is shown in Fig. 4 with the MIC distributions combined from 3 consecutive (2014 to 2016) multinational surveillance studies (20). Regarding the integrated PTA, the highest MIC value achieving >90% PTA was 8 μg/ml for 75% *f*T_>MIC_ regardless of infection site, and it was 4 μg/ml even for 100% *f*T_>MIC_.

**TABLE 3:** Probability of target attainment for 75% *f*T_>MIC_ by infection site and renal function group^*a*^

Renal function group and infection site	Dose regimens with 3-h infusion	PTA (%) for MIC (μg/ml) of:						
		0.25	0.5	1	2	4	8	16
Pneumonia patients								
Augmented renal function	2 g q6h	100	100	100	100	99.7	94.5	60.4
Normal renal function	2 g q8h	100	100	100	99.9	98.9	87.1	43.4
Mild renal impairment	2 g q8h	100	100	100	100	99.8	97.0	69.7
Moderate renal impairment	1.5 g q8h	100	100	100	100	99.9	98.7	83.3
Severe renal impairment	1 g q8h	100	100	100	100	100	99.9	90.7
ESRD	0.75 g q12h	100	100	100	100	100	99.6	86.3
BSI/sepsis patients								
Augmented renal function	2 g q6h	100	100	100	100	99.4	91.3	49.6
Normal renal function	2 g q8h	100	100	100	99.9	97.3	80.6	32.6
Mild renal impairment	2 g q8h	100	100	100	99.9	99.6	94.4	57.7
Moderate renal impairment	1.5 g q8h	100	100	100	100	99.9	98.0	74.8
Severe renal impairment	1 g q8h	100	100	100	100	100	99.8	84.8
ESRD	0.75 g q12h	100	100	100	100	100	99.2	79.2
cUTI patients								
Augmented renal function	2 g q6h	100	100	100	100	99.9	96.9	73.3
Normal renal function	2 g q8h	100	100	100	100	99.6	93.6	56.3
Mild renal impairment	2 g q8h	100	100	100	100	99.8	98.4	81.2
Moderate renal impairment	1.5 g q8h	100	100	100	100	100	99.6	90.4
Severe renal impairment	1 g q8h	100	100	100	100	100	100	95.9
ESRD	0.75 g q12h	100	100	100	100	100	100	91.6

a PK steady state was assumed. Shaded area indicates >90%. There were 1,000 simulated patients in each simulation scenario. Body weight was assumed to be log-normal distributed with mean of 72.6 kg and CV of 30%. Albumin was assumed to be log-normal distributed with mean of 2.8 g/dl and CV of 30%. Augmented, CrCL of >120 ml/min (120 to <150 = 50%; >150 = 50%); normal, CrCL of 90 to <120 ml/min; mild, CrCL of 60 to <90 ml/min; moderate, CrCL of 30 to <60 ml/min; severe, CrCL of 15 to <30 ml/min; ESRD (end-stage renal disease), CrCL of 5 to <15 ml/min.

**TABLE 4:** Probability of target attainment for 100% *f*T_>MIC_ by infection site and renal function group^*a*^

Renal function group and infection site	Dose regimens with 3-h infusion	PTA (%) for MIC (μg/ml) of:						
		0.25	0.5	1	2	4	8	16
Pneumonia patients								
Augmented renal function	2 g q6h	100	100	100	99.7	95.9	79.8	37.0
Normal renal function	2 g q8h	100	100	99.9	98.3	91.2	64.6	23.2
Mild renal impairment	2 g q8h	100	100	99.9	99.7	98.2	85.9	46.4
Moderate renal impairment	1.5 g q8h	100	100	100	100	99.5	94.8	66.7
Severe renal impairment	1 g q8h	100	100	100	100	100	99.5	81.8
ESRD	0.75 g q12h	100	100	100	100	100	98.3	77.1
BSI/sepsis patients								
Augmented renal function	2 g q6h	100	100	100	99.4	93.6	71.6	28.5
Normal renal function	2 g q8h	100	99.9	99.5	96.2	85.8	54.0	14.1
Mild renal impairment	2 g q8h	100	100	99.8	99.4	96.0	78.0	36.1
Moderate renal impairment	1.5 g q8h	100	100	100	99.9	98.7	91.2	55.8
Severe renal impairment	1 g q8h	100	100	100	100	100	98.3	74.7
ESRD	0.75 g q12h	100	100	100	100	100	96.8	68.0
cUTI patients								
Augmented renal function	2 g q6h	100	100	100	100	98.0	88.3	51.1
Normal renal function	2 g q8h	100	100	99.9	99.4	95.1	77.6	34.3
Mild renal impairment	2 g q8h	100	100	100	99.8	98.9	93.2	59.4
Moderate renal impairment	1.5 g q8h	100	100	100	100	99.8	97.7	79.1
Severe renal impairment	1 g q8h	100	100	100	100	100	99.7	90.1
ESRD	0.75 g q12h	100	100	100	100	100	99.4	85.7

a PK steady state was assumed. Shaded area indicates >90%. There were 1,000 simulated patients in each simulation scenario. Body weight was assumed to be log-normal distributed with mean of 72.6 kg and CV of 30%. Albumin was assumed to be log-normal distributed with mean of 2.8 g/dl and CV of 30%. Augmented, CrCL of >120 ml/min (120 to <150 = 50%; >150 = 50%); normal, CrCL of 90 to <120 ml/min; mild, CrCL of 60 to <90 ml/min; moderate, CrCL of 30 to <60 ml/min; severe, CrCL of 15 to <30 ml/min; ESRD (end-stage renal disease), CrCL of 5 to <15 ml/min.

## DISCUSSION

This is an updated report to the previously published research (10, 12) for population PK and PK/PD analyses of cefiderocol by integrating the newly available data in patients with pneumonia, BSI/sepsis, and cUTI caused by Gram-negative pathogens from two phase 3 studies. The updated results based on the target patient population would provide useful information to understand the PK and PK/PD characteristics of cefiderocol in clinical practices.

In the population PK analysis, CrCL was the most significant covariate for cefiderocol PK, as expected, since cefiderocol is mainly excreted via the kidneys (9, 10, 12). The developed model suggested that cefiderocol CL increases following the power model for up to 150 ml/min CrCL with a constant for ≥150 ml/min CrCL. For another cephalosporin, ceftazidime of ceftazidime-avibactam, the relationship between CL and CrCL was assessed using CrCL cutoff value of 100 ml/min in the population PK analysis, and the slope of CL to CrCL for ≥100 ml/min CrCL was found to be much smaller than that for <100 ml/min CrCL (21), which is consistent with the results in this research. The predictability of the Cockcroft-Gault equation for high CrCL (i.e., ≥120 ml/min) is considered low since the number of data points for CrCL ≥120 ml/min was limited for the equation development (8), and the Cockcroft-Gault equation was reported to overestimate renal function for a high CrCL range (22). The low predictability for high CrCL is one of the possible reasons for the relationship between cefiderocol CL and CrCL with two slopes. However, it could be concluded that the cefiderocol PK could be successfully modeled with the developed population PK model, which could be used for subsequent simulation works since the pcVPC plots (as shown in Fig. 1) suggested that the model adequately described the PK profiles of cefiderocol even in patients with augmented renal function.

Augmented renal function, which leads to faster elimination of antibiotics, is observed especially in critically ill patients, e.g., trauma, sepsis, burns, or pancreatitis (23–25). In the phase 3 studies, augmented renal function (defined as CrCL ≥120 ml/min in this study) was observed in 20% of the patients with comparable proportions for CrCL of 120 to <150 and ≥150 ml/min. The estimated free trough concentrations in the phase 3 studies at 2 g every 6 hours (q6h) dosing regimen for the augmented renal function group were higher than 4 μg/ml (minimum, 4.28 μg/ml; geometric mean, 12.7 μg/ml). The geometric mean of estimated daily AUC at 2 g q6h (1,365 μg·h/ml) was similar to that in patients with normal renal function in the phase 3 studies (1,494 μg·h/ml) at 2 g q8h. In addition, Monte-Carlo simulations for augmented renal function at 2 g q6h demonstrated that the PTA for 75% *f*T_>MIC_ was >90% against MICs ≤8 μg/ml regardless of infection site, and even for 100% *f*T_>MIC_, it was >90% against MICs ≤4 μg/ml. These results suggest that the recommended dosing regimen of 2 g q6h over 3 hours infusion would provide sufficient exposures in patients with CrCL of 120 ml/min or greater.

A negative relationship between *V*_1_ and ALB was incorporated in the final model, suggesting larger *V*_1_ in patients with lower ALB. The increase in volume of distribution with hypoalbuminemia is consistent with the report for PK of antibiotics in critically ill patients (23, 24, 26). The estimated *C*_max_ and daily AUC at steady state for the patients in the phase 3 studies were similar between ALB groups (ALB of <2.8 or ≥2.8 g/dl) (Fig. S4 in the supplemental material). Since protein binding data were not available in the subjects used for the analyses, an effect of ALB on the unbound fraction of cefiderocol could not be directly assessed. However, if the unbound fraction was changed depending on albumin concentrations, the total CL of cefiderocol would be changed depending on albumin concentrations since the unbound fraction of cefiderocol is readily excreted via kidney. The fact that there is no clear difference in total CL depending on albumin concentrations in this study suggested the effect of ALB would not be clinically relevant to the exposure to cefiderocol.

The effects of infection sites on CL and *V*_1_ were assessed in the population PK analysis. The CL in patients with cUTI/AUP in the APEKS-cUTI study was 27% higher than that in subjects without infection, which was consistent with the previous analysis (12), while that in patients with pneumonia, BSI/sepsis, and cUTI (CREDIBLE-CR study) was comparable to that in subjects without infection. The *V*_1_ of cefiderocol in the patients with any infection site was 39% higher than that in subjects without infection. These results were consistent with the reports suggesting increased CL and volume of distribution of ceftolozane in cUTI patients (27) and increased volume of distribution of antibiotics in critically ill patients (23, 24, 26). Although the AUC for patients with cUTI/AUP in the APEKS-cUTI study was slightly lower than that for the other patients, including cUTI patients in the CREDIBLE-CR study, the estimated *C*_max_ and AUC overlapped among the infected patients (Fig. 2A). Therefore, the effect of infection sites was considered not to be clinically relevant on the exposure to cefiderocol. The patients’ background characteristics were different among the APEKS-cUTI, CREDIBLE-CR, and APEKS-NP studies, including disease severity and renal/hepatic function as well as the selection of carbapenem-resistant infections in the CREDIBLE-CR study. The effect of infection site could not be distinguished explicitly from these factors in the population PK analyses since they were confounded. In addition, the pathophysiological reason for the effect of the infection site has not been identified, which is the limitation of the developed population PK model.

There have been reports for a changed volume of distribution of antibiotics in patients with mechanical ventilation, although the estimated influences were variable (21, 23, 28). The volume of distribution of ceftazidime in nosocomial pneumonia patients with ventilation (NPv) was 30% higher than that in non-NPv patients (21), while the *V*_1_ of ceftazidime in intensive care unit patients with ventilation was about half of that in patients without ventilation (28). As for cefiderocol, mechanical ventilation was not a significant covariate on CL or *V*_1_. In addition, the estimated *C*_max_ and AUC were similar between the pneumonia patients with and without mechanical ventilation in the phase 3 studies (Fig. 2B). Therefore, it could be concluded that the effect of ventilation would not be clinically relevant to cefiderocol PK.

The %*f*T_>MIC_ was 100% in 97% of the patients in the phase 3 studies, suggesting adequate exposure to cefiderocol was achieved against MIC of causative Gram-negative pathogens (≤0.03 to 64 μg/ml; MIC_90_ of 2 μg/ml in both CREDIBLE-CR and APEKS-NP studies). The geometric means (range) of estimated free trough concentrations were 16.3 (2.91 to 84.8) μg/ml in the CREDIBLE-CR study and 12.7 (0.856 to 89.5) μg/ml in the APEKS-NP study. Based on the Monte-Carlo simulation, the PTA for 75% *f*T_>MIC_ was >95% against MICs ≤4 μg/ml regardless of infection site or renal function. The PTA even for 100% *f*T_>MIC_ was >90% against MICs ≤4 μg/ml for all of the infection sites and renal function groups except for the normal renal function in BSI/sepsis patients (85%). For PTA calculations, the target 75% *f*T_>MIC_ was selected as the mean value achieving a bactericidal effect (1 log_10_ reduction) in animal infection models, and 100% *f*T_>MIC_ was used as a very conservative target in consideration with variations in the estimated %*f*T_>MIC_ among pathogens in animal infection models (5). In 3 multinational surveillance studies (SIDERO-WT-2014/2015/2016), cefiderocol suppressed the growth of ≥97.0% of meropenem-nonsusceptible strains of *Enterobacteriaceae*, ≥99.7% of P. aeruginosa, ≥91.0% of A. baumannii, and ≥99.4% of S. maltophilia at ≤4 μg/ml (20). These studies also support that cefiderocol has antibacterial activity against more than 90% of meropenem-nonsusceptible strains with MICs of ≤4 μg/ml, and the recommended dose regimens would provide sufficient exposure against their causative pathogens.

No clear PK/PD relationship was found for any of the outcomes or vital status, which was because the %*f*T_>MIC_ was 100% in most of the patients in the phase 3 studies. The eradication rates were 33% to 44%, even at 100% *f*T_>MIC_ in the phase 3 studies. Most microbiological outcomes were indeterminate. Microbiological eradication in a population as complex as that enrolled in the phase 3 studies is often confounded by nonstudy antibiotics, missing data, and the continued presence of foreign body devices such as endotracheal tubes. For critically ill patients, higher target concentrations (e.g., 4-fold MIC) were considered a PK/PD index as reported for β-lactams (15). Even for 4-fold MIC as a target, %*f*T_>MIC_ (%*f*T_>4×MIC_) was 100% in 83% of the patients in the phase 3 studies and no PK/PD relationships with %*f*T_>4×MIC_ were found (data are not shown).

In the CREDIBLE-CR study, the estimated *C*_max_ and daily AUC of cefiderocol in death cases (*n* = 18) were 1.4-fold of those in survival cases (*n* = 54) (Fig. S5). Preclinical concentration-dependent toxicology studies suggest that the no-observed-adverse-effect level of exposure is 9-fold of that achieved at the standard dosing regimen (2 g q8h) (29). The AUC values in two death cases were less than 2-fold of the maximum AUC in survival cases in the CREDIBLE-CR study, and they did not reach the level that might be associated with increased risk of toxicity based on toxicology studies (29). The causes of death in the two patients were considered to be related to exacerbation of the underlying illness and infection and/or were complicated with a history of shock within 31 days at the time of randomization (18).

Epithelial lining fluid (ELF) is an important consideration for the treatment of patients with pneumonia. In a very recent study, ELF concentrations were determined from mechanically ventilated patients with bacterial pneumonia (30), and ELF PTA was calculated based on these data as well as ELF concentrations in healthy subjects (31) using intrapulmonary PK modeling (32). The results suggested that sufficient drug exposures could be achieved in ELF in all renal function groups for 100% *f*T_>MIC_ for an MIC of ≤4 μg/ml.

The breakpoints of cefiderocol are inconsistent among the agency/organization (FDA, CLSI, and EUCAST) (33; https://www.fda.gov/drugs/development-resources/antibacterial-susceptibility-test-interpretive-criteria; http://www.eucast.org/clinical_breakpoints/). For example, the breakpoints for *Enterobacterales* determined by FDA and CLSI are 4/8/16 μg/ml (susceptible/intermediate/resistant), while those by EUCAST are 2/4 μg/ml (susceptible/resistant). More details were discussed by Simner et al. and Yamano et al. (34, 35).

In summary, the developed population PK model adequately described plasma cefiderocol concentrations in subjects without infection and patients with pneumonia, BSI/sepsis, and cUTI/AUP. CrCL was the most significant covariate on cefiderocol PK. In the phase 3 studies, the %*f*T_>MIC_ was 100% in almost all of the patients (97%), including the patients with augmented renal function, ventilation, and/or were critically ill in the intensive care unit. Adequate plasma exposure to cefiderocol can be achieved at the recommended dosing regimen of 2 g q8h over 3 hours infusion and the regimens adjusted based on renal function in patients with pneumonia, BSI/sepsis, or cUTI caused by Gram-negative pathogens, including carbapenem-resistant strains.

## MATERIALS AND METHODS

### Data for analyses.

Plasma cefiderocol concentration data were collected from 115 pneumonia patients in the APEKS-NP study (https://www.clinicaltrials.gov identifier NCT03032380) (19), 72 patients with pneumonia, BSI/sepsis, or cUTI in the CREDIBLE-CR study (https://www.clinicaltrials.gov identifier NCT02714595) (18), 238 patients with cUTI/AUP in the APEKS-cUTI study (https://www.clinicaltrials.gov identifier NCT02321800) (17), and 91 subjects without any infection in phase 1 studies (9, 36) as shown in Table S1 in the supplemental material. The pneumonia patients enrolled in the APEKS-NP and CREDIBLE-CR studies included patients with hospital-acquired pneumonia, ventilator-associated pneumonia, and health care-associated pneumonia. Population PK models of cefiderocol were previously developed using the data without phase 3 studies (10, 12). In this study, a population PK model was developed using the updated data set with additional data from patients with pneumonia, BSI/sepsis, or cUTI caused by Gram-negative pathogens, including carbapenem-resistant pathogens.

Plasma concentration data for 32 patients who received hemodialysis in the phase 3 studies were excluded from the analysis. Six concentration data values in the phase 3 studies were considered to be anomalous and excluded from the analysis since they were approximately 10-fold higher than the *C*_max_ following a 2-g single dose infused over 3 hours in a phase 1 study. There were 363 plasma concentrations below the limit of quantification (BLQ) excluded from the analysis. In phase 1 studies, most BLQ data (334 out of 353 plasma concentrations) were predose or ≥24 hours postdose when plasma concentrations had been expected to be zero or very low. The rest of the 19 BLQ data were 12 to 24 hours postdose at lower doses of 100, 250, and 500 mg. In the CREDIBLE-CR and APEKS-cUTI studies, data of 9 plasma concentrations in 3 patients (data from 3 plasma concentrations each) were BLQ at all sampling points, which were considered to be anomalous. In the APEKS-NP study, BLQ data were observed at 1 point (just prior to the start of infusion). This exclusion of BLQ data would not affect the results of modeling because the developed model predicted plasma concentrations reasonably for any study and renal function group (Fig. 1). The detail for the excluded data in the APEKS-cUTI and phase 1 studies were described in the previous reports (10, 12).

Consequently, the population PK model was developed using a total of 3,427 plasma concentrations from 516 subjects, 1,861 plasma concentrations from 91 uninfected subjects, and 1,566 plasma concentrations from 425 patients with infection (Fig. S1). The PK/PD analysis was conducted in the patients who had data for MIC of causative Gram-negative pathogens and microbiological or clinical outcomes following cefiderocol dosing, 60 patients in the CREDIBLE-CR study, and 97 patients in the APEKS-NP study. The total numbers of isolated pathogens at baseline in the CREDIBLE-CR and APEKS-NP studies were 77 and 122, respectively.

### Bioanalytical method.

A bioanalytical method for the determination of plasma total cefiderocol concentrations was validated where the lower limit of quantification was 0.1 μg/ml (9). Composite plasma samples mixed with 0.2 mol/liter ammonium acetate (pH 5) in a 1:1 volume ratio were prepared and analyzed by a validated liquid chromatography-tandem mass spectrometry assay. The assay was linear from 0.1 to 100 μg/ml, and the precision and accuracy levels were 1.2% to 13.4% and −7.0% to 7.0%, respectively.

### Population pharmacokinetic analysis.

A 3-compartment model was initially tested as a structural PK model based on the previous analysis (10, 12). An interindividual variability for PK parameters was assumed to follow a log-normal distribution and could be modeled with an exponential error model. A model for intraindividual variability was selected from a proportional error model or a combined error model (additive error plus proportional error model).

A covariate model was developed to identify influencing covariates on cefiderocol PK. The effects of the following covariates on CL were tested; CrCL was calculated by the Cockcroft-Gault equation (8), body weight, age, sex, aspartate aminotransferase, alanine aminotransferase, total bilirubin, ALB, race, infection site (no infection, infection with pneumonia in the APEKS-NP and CREDIBLE-CR studies, infection with BSI/sepsis, infection with cUTI in the CREDIBLE-CR study, or infection with cUTI/AUP in the APEKS-cUTI study), and ventilation (mechanical ventilation during PK sampling). Age, sex, ALB, race, infection site, and ventilation were also tested as covariates on *V*_1_, and body weight was tested as a covariate on *V*_1_, *V*_2_, and intercompartmental clearance (*Q*_2_).

The effect of CrCL on CL was initially tested using a power model, a piecewise linear model, and a power plus linear combination model with a CrCL cutoff value of 150 ml/min based on the visual inspection of the relationship between CL and CrCL (Fig. S3). The power plus linear combination model was selected based on OBJ, and the slope of CL to CrCL for CrCL of ≥150 ml/min was extremely small (<0.0001). Therefore, the models in which CL was assumed to be constant for CrCL values of ≥120, 150, or 180 ml/min were tested, and the CrCL cutoff value of 150 ml/min was selected based on the model fitting assessed by OBJ. Next, the effect of body weight on the PK parameters was tested based on the physiological aspect. After incorporating CrCL and body weight into the model, the other covariates were tested using a univariate regression analysis as screening. The significance level of 0.01 based on χ^2^ test (*P* < 0.01) was used for inclusion of covariates into the model.

After incorporating all covariates which were statistically significant in the screening, an inferential assessment and stepwise backward deletion were performed to refine the model. In the inferential assessment, the ratio of parameters and the 95% confidence interval were calculated based on the parameter estimate and standard error and compared with a clinically insignificant range, 0.80 to 1.25, to evaluate the impact of covariate effect. In the stepwise backward deletion, the significance level of 0.001 based on χ^2^ test (*P* < 0.001) was used for construction of a final model.

The developed population PK model was evaluated based on GOF plots. The predictive performance was also evaluated by the pcVPC (37) with 500 simulation runs. In addition, the model robustness was evaluated by a bootstrap technique (38). Resampling from the original data set was conducted for generating 300 bootstrap data sets, and PK parameters were estimated for each of the data sets using the final model. The median and 95% CI of the bootstrap estimates were compared to the parameter estimate for the final model.

### Pharmacokinetic/pharmacodynamic analysis.

The *C*_max_ and daily AUC for infected patients were calculated using *post hoc* PK parameters for the final model. Individual %*f*T_>MIC_ was calculated based on the MIC of causative Gram-negative pathogens, and the simulated steady-state free plasma concentrations were calculated using an unbound fraction of 0.422 (16).

Relationships of %*f*T_>MIC_ with microbiological outcome and clinical outcome at test of cure and vital status on day 28 from the start of treatment were evaluated using data in the phase 3 studies. The data of “eradication” for microbiological outcome, “clinical cure” for clinical outcome, and “survival” for vital status were treated as positive outcomes. The data of “persistence” and “indeterminate” for microbiological outcome, “clinical failure” and “indeterminate” for clinical outcome, and “death” for vital status were treated as negative outcomes. In cases where more than one causative pathogens were detected, the pathogen with highest MIC was used to evaluate the relationships with clinical outcome and vital status, while the MICs of each pathogen were used to evaluate the relationship with microbiological outcome.

### Monte-Carlo simulations.

Monte-Carlo simulations were performed to calculate PTA for 75% *f*T_>MIC_ and 100% *f*T_>MIC_ for patients with pneumonia, BSI/sepsis, and cUTI. The simulation for cUTI patients was performed using the parameters for cUTI in the CREDIBLE-CR study to assess the PTA for the target %*f*T_>MIC_ in critically ill patients with infection caused by carbapenem-resistant Gram-negative pathogens. A thousand virtual patients for each infection site (pneumonia, BSI/sepsis, or cUTI) were generated by simulating CrCL, body weight, and ALB, which were significant covariates in the population PK analysis. The PTA was calculated by infection site and renal function group. The integrated PTA was also calculated by weighting proportions of patients in each renal function group based on the distribution of CrCL in the phase 3 studies (CrCL of ≥120 ml/min, 20.3%; CrCL of 90 to <120 ml/min, 15.0%; CrCL of 60 to <90 ml/min, 24.6%; CrCL of 30 to <60 ml/min, 32.6%; CrCL of 15 to <30 ml/min, 4.8%l CrCL of 5 to <15 ml/min, 2.7%). The %*f*T_>MIC_ was calculated against an MIC range of 0.25 to 16 μg/ml. The dose regimen for the simulation was set as follows: 2 g q6h for augmented renal function with ≥120 ml/min CrCL, 2 g q8h for normal renal function and mild renal impairment with CrCL of 60 to <120 ml/min, 1.5 g q8h for moderate renal impairment with CrCL of 30 to <60 ml/min, 1 g q8h for severe renal impairment with CrCL of 15 to <30 ml/min, and 0.75 g q12h for end-stage renal disease (ESRD) with CrCL of 5 to <15 ml/min.

### Software.

Model-building and Monte-Carlo simulations were performed using NONMEM (version 7.3.0) (39), Perl-speaks NONMEM (version 4.2.0) (40, 41), and Pirana (version 2.9.4) (41). R (version 3.5.1) (42) was used to calculate *post hoc* PK parameters and PTA.

## Supplementary Material

Supplemental file 1

## References

[1] Kohira N, West J, Ito A, Ito-Horiyama T, Nakamura R, Sato T, Rittenhouse S, Tsuji M, Yamano Y. 2015. In vitro antimicrobial activity of a siderophore cephalosporin, S-649266, against Enterobacteriaceae clinical isolates, including Carbapenem-resistant strains. Antimicrob Agents Chemother 60:729–734. doi:10.1128/AAC.01695-15. 26574013 PMC4750680

[2] Ito A, Kohira N, Bouchillon SK, West J, Rittenhouse S, Sader HS, Rhomberg PR, Jones RN, Yoshizawa H, Nakamura R, Tsuji M, Yamano Y. 2016. In vitro antimicrobial activity of S-649266, a catechol-substituted siderophore cephalosporin, when tested against non-fermenting Gram-negative bacteria. J Antimicrob Chemother 71:670–677. doi:10.1093/jac/dkv402. 26645269

[3] Ito A, Sato T, Ota M, Takemura M, Nishikawa T, Toba S, Kohira N, Miyagawa S, Ishibashi N, Matsumoto S, Nakamura R, Tsuji M, Yamano Y. 2017. In vitro antibacterial properties of cefiderocol, a novel siderophore cephalosporin, against Gram-negative bacteria. Antimicrob Agents Chemother 62:e01454-17. doi:10.1128/AAC.01454-17. 29061741 PMC5740388

[4] Sato T, Yamawaki K. 2019. Cefiderocol: discovery, chemistry, and in vivo profiles of a novel siderophore cephalosporin. Clin Infect Dis 69(Suppl 7):S538–S543. doi:10.1093/cid/ciz826. 31724047 PMC6853759

[5] Nakamura R, Ito-Horiyama T, Takemura M, Toba S, Matsumoto S, Ikehara T, Tsuji M, Sato T, Yamano Y. 2019. In vivo pharmacodynamic study of cefiderocol, a novel parenteral siderophore cephalosporin, in murine thigh and lung infection models. Antimicrob Agents Chemother 63:e02031-18. doi:10.1128/AAC.02031-18. 31262762 PMC6709502

[6] Shionogi Inc. 2020. Fetroja (cefiderocol) prescribing information. Shionogi Inc., Florham Park, NJ.

[7] European Medicines Agency. 2020. Fetcroja (cefiderocol) product information. European Medicines Agency, Amsterdam, The Netherlands. https://www.ema.europa.eu/documents/product-information/fetcroja-epar-product-information_en.pdf

[8] Cockcroft DW, Gault MH. 1976. Prediction of creatinine clearance from serum creatinine. Nephron 16:31–41. doi:10.1159/000180580. 1244564

[9] Katsube T, Echols R, Arjona Ferreira JC, Krenz HK, Berg JK, Galloway C. 2017. Cefiderocol, a siderophore cephalosporin for Gram-negative bacterial infections: pharmacokinetics and safety in subjects with renal impairment. J Clin Pharmacol 57:584–591. doi:10.1002/jcph.841. 27874971 PMC5412848

[10] Katsube T, Wajima T, Ishibashi T, Arjona Ferreira JC, Echols R. 2016. Pharmacokinetic/pharmacodynamic modeling and simulation of cefiderocol, a parenteral siderophore cephalosporin, for dose adjustment based on renal function. Antimicrob Agents Chemother 61:e01381-16. doi:10.1128/AAC.01381-16. 27795374 PMC5192099

[11] Katsube T, Echols R, Wajima T. 2019. Pharmacokinetics and pharmacodynamic profiles of cefiderocol, a novel siderophore cephalosporin. Clin Infect Dis 69(Suppl 7):S552–S558. doi:10.1093/cid/ciz828. 31724042 PMC6853762

[12] Kawaguchi N, Katsube T, Echols R, Wajima T. 2018. Population pharmacokinetic analysis of cefiderocol, a parenteral siderophore cephalosporin, in healthy subjects, subjects with various degrees or renal function, and patients with complicated urinary tract infection of acute uncomplicated pyelonephritis. Antimicrob Agents Chemother 62:e01391-17. doi:10.1128/AAC.01391-17. 29038272 PMC5786804

[13] Craig WA. 1998. Pharmacokinetic/pharmacodynamic parameters: rationale for antibacterial dosing of mice and men. Clin Infect Dis 26:1–12. doi:10.1086/516284. 9455502

[14] Andes D, Craig WA. 2002. Animal model pharmacokinetics and pharmacodynamics: a critical review. Int J Antimicrob Agents 19:261–268. doi:10.1016/S0924-8579(02)00022-5. 11978497

[15] Goncalves-Pereira J, Povoa P. 2011. Antibiotics in critically ill patients: a systematic review of the pharmacokinetics of β-lactams. Crit Care 15:R206. doi:10.1186/cc10441. 21914174 PMC3334750

[16] Matsumoto S, Singley CM, Hoover J, Nakamura R, Echols R, Rittenhouse S, Tsuji M, Yamano Y. 2017. Efficacy of cefiderocol against Carbapenem-resistant Gram-negative Bacilli in immunocompetent-rat respiratory tract infection models recreating human plasma pharmacokinetics. Antimicrob Agents Chemother 61:e00700-17. doi:10.1128/AAC.00700-17. 28630178 PMC5571323

[17] Portsmouth S, van Veenhuyzen D, Echols R, Machida M, Arjona Ferreira JC, Ariyasu M, Tenke P, Nagata TD. 2018. Cefiderocol versus imipenem-cilastatin for the treatment of complicated urinary tract infections caused by Gram-negative uropathogens: a phase 2, randomized, double-blind, non-inferiority trial. Lancet Infect Dis 18:1319–1328. doi:10.1016/S1473-3099(18)30554-1. 30509675

[18] Bassetti M, Echols R, Matsunaga Y, Ariyasu M, Doi Y, Ferrer R, Lodise TP, Naas T, Niki Y, Paterson DL, Portsmouth S, Torre-Cisneros J, Toyoizumi K, Wunderink RG, Nagata TD. 2020. Efficacy and safety of cefiderocol for the treatment of serious infections caused by carbapenem-resistant Gram-negative bacteria (CREDIBLE-CR): results of a phase 3 randomised, open-label, parallel-assigned, pathogen-focused study. Lancet Infect Dis, in press. doi:10.1016/S1473-3099(20)30796-9. 33058795

[19] Wunderink RG, Matsunaga Y, Ariyasu M, Clevenbergh P, Echols R, Kaye KS, Kollef M, Menon A, Pogue JM, Shorr AF, Timsit JF, Zeitlinger M, Nagata TD. 2020. Cefiderocol versus high-dose, extended-infusion meropenem for the treatment of Gram-negative nosocomial pneumonia (APEKS-NP): a phase 3, randomised, double-blind, non-inferiority study. Lancet Infect Dis, in press. doi:10.1016/S1473-3099(20)30731-3. 33058798

[20] Yamano Y. 2019. In vitro activity of cefiderocol against a broad range of clinically important Gram-negative bacteria. Clin Infect Dis 69(Suppl 7):S544–S551. doi:10.1093/cid/ciz827. 31724049 PMC6853761

[21] Li J, Lovern M, Green ML, Chiu J, Zhou D, Comisar C, Xiong Y, Hing J, MacPherson M, Wright JG, Riccobene T, Carrothers TJ, Das S. 2019. Ceftazidime-avibactam population pharmacokinetic modeling and pharmacodynamic target attainment across adult indications and patient subgroups. Clin Transl Sci 12:151–163. doi:10.1111/cts.12585. 30221827 PMC6440567

[22] Grootaert V, Willems L, Debaveye Y, Meyfroidt G, Spriet I. 2012. Augmented renal clearance in the critically ill: how to assess kidney function. Ann Pharmacother 46:952–959. doi:10.1345/aph.1Q708. 22693271

[23] Roberts JA, Abdul-Aziz MH, Lipman J, Mouton JW, Vinks AA, Felton TW, Hope WW, Farkas A, Neely MN, Schentag JJ, Drusano G, Frey OR, Theuretzbacher U, Kuti JL. 2014. Individualised antibiotic dosing for patients who are critically ill: challenges and potential solutions. Lancet Infect Dis 14:498–509. doi:10.1016/S1473-3099(14)70036-2. 24768475 PMC4181663

[24] Blot SI, Pea F, Lipman J. 2014. The effect of pathophysiology on pharmacokinetics in the critically ill patient – concepts appraised by the example of antimicrobial agents. Adv Drug Deliv Rev 77:3–11. doi:10.1016/j.addr.2014.07.006. 25038549

[25] Mahmoud SH, Shen C. 2017. Augmented renal clearance in critical illness: an important consideration in drug dosing. Pharmaceutics 9:E36. doi:10.3390/pharmaceutics9030036. 28926966 PMC5620577

[26] Ulldemolins M, Roberts JA, Rello J, Paterson DL, Lipman J. 2011. The effects of hypoalbuminaemia on optimizing antibacterial dosing in critically ill patients. Clin Pharmacokinet 50:99–110. doi:10.2165/11539220-000000000-00000. 21142293

[27] Chandorkar G, Xiao A, Mouksassi MS, Hershberger E, Krishna G. 2015. Population pharmacokinetics of ceftolozane/tazobactam in healthy volunteers, subjects with varying degrees of renal function and patients with bacterial infections. J Clin Pharmacol 55:230–239. doi:10.1002/jcph.395. 25196976 PMC4303958

[28] Georges B, Conil JM, Seguin T, Ruiz S, Minville V, Cougot P, Decun JF, Gonzalez H, Houin G, Fourcade O, Saivin S. 2009. Population pharmacokinetics of ceftazidime in intensive care unit patients: influence of glomerular filtration rate, mechanical ventilation, and reason for admission. Antimicrob Agents Chemother 53:4483–4489. doi:10.1128/AAC.00430-09. 19635962 PMC2764213

[29] Sanabria C, Migoya E, Mason JW, Stanworth SH, Katsube T, Machida M, Narukawa Y, Nagata TD. 2019. Effect of cefiderocol, a siderophore cephalosporin, on QT/QTc interval in healthy adult subjects. Clin Ther 41:1724–1736. doi:10.1016/j.clinthera.2019.07.006. 31378318

[30] Katsube T, Wajima T, Echols R, Portsmouth S, Ariyasu M, Rodvold K, Nicolau DP. 2019. Intrapulmonary pharmacokinetics of cefiderocol in hospitalized and ventilated patients receiving standard of care antibiotics for bacterial pneumonia. Abstr IDWeek 2020, San Diego, CA.

[31] Katsube T, Saisho Y, Shimada J, Furuie H. 2019. Intrapulmonary pharmacokinetics of cefiderocol, a novel siderophore cephalosporin, in healthy adult subjects. J Antimicrob Chemother 74:1971–1974. doi:10.1093/jac/dkz123. 31220260 PMC6587409

[32] Katsube T, Kawaguchi N, Echols R, Wajima T, Nicolau DP. 2020. Cefiderocol population pharmacokinetics and probability of target attainment in plasma and epithelial lining fluid in patients with pneumonia, bloodstream infection/sepsis, or complicated urinary tract infections. Abstr IDWeek 2020, San Diego, CA.

[33] Clinical and Laboratory Standards Institute. 2020. Performance standards for antimicrobial susceptibility testing; thirtieth informational supplement. CLSI M100-S30. Clinical and Laboratory Standards Institute, Wayne, PA.

[34] Simner PJ, Patel R. 2020. Cefiderocol antimicrobial susceptibility testing considerations: the Achilles heel of the Trojan horse? J Clin Microbiol, in press. doi:10.1128/JCM.00951-20. PMC7771437 32727829

[35] Yamano Y, Takemura M, Longshaw C, Echols R. 2020. Differences in interpretative breakpoints between CLSI, FDA and EUCAST impact reporting of susceptibility and resistance to cefiderocol. Abstr IDWeek 2020, San Diego, CA.

[36] Saisho Y, Katsube T, White S, Fukase H, Shimada J. 2018. Pharmacokinetics, safety, and tolerability of cefiderocol, a novel siderophore cephalosporin for Gram-negative bacteria, in healthy subjects. Antimicrob Agents Chemother 62:e02163-17. doi:10.1128/AAC.02163-17. 29311072 PMC5826143

[37] Bergstrand M, Hooker AC, Wallin JE, Karlsson MO. 2011. Prediction-corrected visual predictive checks for diagnosing nonlinear mixed-effects models. AAPS J 13:143–151. doi:10.1208/s12248-011-9255-z. 21302010 PMC3085712

[38] Ette EI. 1997. Stability and performance of a population pharmacokinetic model. J Clin Pharmacol 37:486–495. doi:10.1002/j.1552-4604.1997.tb04326.x. 9208355

[39] Beal SL, Sheiner LB, Boeckmann AJ. 2006. NONMEM users guide. Icon Development Solutions, Ellicott City, MD.

[40] Lindbom L, Pihlgren P, Jonsson EN. 2005. PsN-Toolkit – a collection of computer intensive statistical methods for non-linear mixed effect modeling using NONMEM. Comput Methods Programs Biomed 79(3): 241–57. doi:10.1016/j.cmpb.2005.04.005. 16023764

[41] Keizer RJ, Karlsson MO, Hooker A. 2015. Modeling and simulation workbench for NONMEM: tutorial on Pirana, PsN, and Xpose. CPT Pharmacometrics Syst Pharmacol 2:e50. doi:10.1038/psp.2013.24. PMC3697037 23836189

[42] R Core Team. 2013. R: a language and environment for statistical computing. R Foundation for Statistical Computing, Vienna, Austria.

