# Supplementary material for: Population Pharmacokinetic and Pharmacokinetic/Pharmacodynamic Analyses of Cefiderocol, a Parenteral Siderophore Cephalosporin, in Patients with Pneumonia, Bloodstream Infection/Sepsis, or Complicated Urinary Tract Infection
Source: Antimicrob Agents Chemother. 2021 Feb 17;65(3):e01437-20. doi: 10.1128/AAC.01437-20 (PMC8092503; doi:10.1128/AAC.01437-20)

Supplemental Materials

Table S1 Summary of Clinical Study Designs

Study	Dose regimen	Subjects dosed cefiderocol	Plasma PK sampling
Phase 1 single and multiple ascending dose study for healthy subjects in Japan	Part 1:<br>Single cefiderocol 0.1-, 0.25-, 0.5-, 1-, 2-g doses or matching placebo infused over 1 hour<br><br>Part 2:<br>Multiple cefiderocol 1- or 2-g doses, or matching placebo infused over 1 hour q8h for 10 days	Part 1: 30<br><br>Part 2: 24	Part 1:<br>Predose, 0.5, 1, 1.25, 1.5, 2, 2.5, 3, 3.5, 4, 4.5, 5, 6, 8, 10, 12, 16, 24, 36 and 48 hours from the start of the infusion<br><br>Part 2:<br>Day 1 (morning dose: first dose): predose, 0.5, 1, 1.25, 1.5, 2, 2.5, 3, 3.5, 4, 4.5, 5, 6, 8, 10, 12 and 16 hours<br>Days 2, 3, 5, 8 and 9 (each morning dose): predose<br>Day 10 (morning dose: last dose): predose, 0.5, 1, 1.25, 1.5, 2, 2.5, 3, 3.5, 4, 4.5, 5, 6, 8, 10, 12, 16, 24, 36 and 48 hours from the start of the infusion
Phase 1 renal impairment study in US	Single cefiderocol 1-g dose infused over 1 hour	38;<br>8 subjects each: normal renal function, mild and moderate renal impairment and ESRD requiring hemodialysis; 6 subjects: severe renal impairment	Predose, 0.25, 0.5, 1, 1.5, 2, 3, 4, 5, 6, 8, 12, 16, 24, 36, 48 and 72 hours from the start of the infusion
Phase 2 APEKS-cUTI study for patients with cUTI and AUP (NCT02321800)	Cefiderocol 2-g doses infused over 1 hour q8h with adjustments for creatinine clearance and body size	300	Just prior to the infusion of the dose, −0.25 to 0 hours at the end of infusion, and 1 ± 0.5 hours after the end of infusion on Day 3
Phase 3 CREDIBLE-CR study for patients with pneumonia, BSI/sepsis, or cUTI (NCT02714595)	Cefiderocol 2-g doses infused over 3 hour q8h with adjustments for eGFR and creatinine clearance	101	Just prior to the start of infusion, 1 hour after the start of infusion, at the end of infusion, and 1 hour after the end of infusion on Day 3.<br>For patients with nonstable renal function resulting in a dosing adjustment, another PK sampling was performed within 24 to 72 hours after their dosing adjustment at the same timing on Day 3.
Phase 3 APEKS-NP study for patients with	Cefiderocol 2-g doses infused over 3 hour q8h with	148	Just prior to the start of infusion, 1 hour after the start of infusion, before

pneumonia (NCT03032380)	adjustments for eGFR and creatinine clearance		the end of infusion, and 1 hour after the end of infusion on Day 3 or Day 4. For patients with nonstable renal function resulting in a dosing adjustment, another PK sampling was performed within 24 to 72 hours after their dosing adjustment at the same timing as above.

Table S2 NONMEM Control File for the Final Model

```
$PROBLEM    Cefiderocol Plasma in FTIH, Renal imp, cUTI, CR, and NP Studies
$INPUT    ID DAT2=DROP TIME TIME2 TAD TAD2 DAY TN AMT DI TINF TINFF RATE EVID
MDV DV AGE SEX RACE WHT HT WT BSA
          ALB ALT AST BIL EGFR EGFR2 CLCR TEGFR TEGFR2 TCLCR PT PTNP APACHE
SOFA VITAL SEVERE HD VENT UID=DROP DGR EXCL SHIP ANL
$DATA      ppkdata_crnp.csv IGNORE=@ IGNORE=(ANL.EQ.1)
$SUBROUTINES    ADVAN = ADVAN11      ;      Three compartment Model
$SUBROUTINES    TRANS = TRANS4
$PK
      IF (ID.LT.2000.AND.CLCR.LT.150) THEN
      CLCR1 = (CLCR/83.0)**THETA(8)
      ENDIF
      IF (ID.GE.2000.AND.TCLCR.LT.150) THEN
      CLCR1 = (TCLCR/83.0)**THETA(8)
      ENDIF
      IF (ID.LT.2000.AND.CLCR.GE.150) THEN
      CLCR1 = (150/83.0)**THETA(8)
      ENDIF
      IF (ID.GE.2000.AND.TCLCR.GE.150) THEN
      CLCR1 = (150/83.0)**THETA(8)
      ENDIF

      WT1 = (WT/72.6)**THETA(9)

      IF (PT.EQ.1.AND.ID.LT.2000) THEN
      NPT11 = 1 ;cUTI/AUP in Ph2
      ELSE
      NPT11 = 0 ;except for cUTI/AUP in Ph2
      ENDIF
      PT11 = THETA(10)**NPT11

      IF (PT.EQ.1.AND.ID.GE.2000) THEN
      NPT12 = 1 ;cUTI in Ph3 CR
      ELSE
```

```
NPT12 = 0 ;except for cUTI in Ph3 CR
ENDIF
PT12 = THETA(11)**NPT12

IF (PT.EQ.3) THEN
NPT13 = 1 ;BSI in Ph3 CR
ELSE
NPT13 = 0 ;except for BSI in Ph3 CR
ENDIF
PT13 = THETA(12)**NPT13

IF (PT.EQ.2) THEN
NPT14 = 1 ;Pneumonia
ELSE
NPT14 = 0 ;except for Pneumonia
ENDIF
PT14 = THETA(13)**NPT14

IF (PT.GE.1) THEN
NPT = 1 ;infection
ELSE
NPT = 0 ;except for infection
ENDIF
PTV = THETA(15)**NPT

ALB2 = (ALB/3.9)**THETA(14)

TVCL = THETA(1)*CLCR1 *PT11 *PT12 *PT13 *PT14
TVV1 = THETA(2)*WT1 *ALB2 *PTV
TVQ2 = THETA(3)
TVV2 = THETA(4)*WT1
TVQ3 = THETA(5)
TVV3 = THETA(6)
;
CL    = TVCL * EXP(ETA(1))
V1    = TVV1 * EXP(ETA(2))
```

```
        Q2    = TVQ2
        V2    = TVV2 * EXP(ETA(3))
        Q3    = TVQ3
        V3    = TVV3
;
        S1      = V1                  ; Scale parameter (S1)

$ERROR
        IPRED = F
        IRES = DV - IPRED
        W = IPRED* THETA(7)
        IF(W.EQ.0) W=1
        IWRES = IRES/W
        Y = IPRED + W*EPS(1)
;
$THETA (0, 4.0, 10.0)    ;    CL    (L/hr)
$THETA (0, 10.0, 20.0)    ;    V1    (Liter)
$THETA (0, 6, 20.0)    ;    Q2 (L/hr)
$THETA (0, 5, 20.0)    ;    V2 (Liter)
$THETA (0, 0.1, 1.0)    ;    Q3 (L/hr)
$THETA (0, 0.8, 2.0)    ;    V3 (Liter)
$THETA (0, 0.1, 1.0)    ;    SIGMA1
$THETA (0, 0.6, 2.0) ; CLCR
$THETA (0, 0.5,2.0) ; WT on V1 and V2
$THETA    (0,1.0,3.0) ; cUTI/AUP in ph2 on CL
$THETA    (0,0.9,2.0) ; cUTI in ph3 CR study on CL
$THETA    (0,1.0,2.0) ; BSI in ph3 CR study on CL
$THETA    (0,1.0,2.0) ; Pneumonia
$THETA (-2.0,-0.5,0.5); ALB on V1
$THETA    (0,1.3,2.0) ; infection
;
$OMEGA BLOCK(3)
0.1
0.1 0.2
0.05 0.1 0.1
$SIGMA 1 FIX
```

```
;
$EST MAXEVAL=9990 PRINT=5 NOABORT METHOD=1 INTERACTION
$COV
$TABLE ONEHEADER NOPRINT FILE=finalmodel.tab
    ID TIME TIME2 TAD TAD2 DAY TN AMT DI TINF TINFF RATE EVID MDV DV AGE SEX
RACE WHT WT
    ALB ALT AST BIL EGFR EGFR2 CLCR TCLCR PT PTNP APACHE SOFA VITAL SEVERE
HD VENT DGR
    CL V1 Q2 V2 Q3 V3 ETA1 ETA2 ETA3 IPRED CWRES CWRESI IRES IWRES
```

Table S3 MIC Distribution for Each Pathogen in CREDIBLE-CR Study (A) and APEKS-NP Study (B)

(A)

	Number of pathogen by MIC									
	MIC (μg/mL)									
**Pathogen**	≤ 0.03	0.06	0.12	0.25	0.5	1	2	4	16	64
ACINETOBACTER BAUMANNII	0	4	5	7	2	5	1	1	1	0
ACINETOBACTER NOSOCOMIALIS	0	0	0	0	0	0	0	0	0	1
ACINETOBACTER RADIORESISTENS	0	0	0	0	0	0	0	0	0	0
CHRYSEOBACTERIUM INDOLOGENES	0	0	0	0	0	1	0	0	0	0
ENTEROBACTER CLOACAE	0	0	0	0	0	0	0	0	1	0
ESCHERICHIA COLI	1	0	0	1	1	0	0	0	1	0
KLEBSIELLA OXYTOCA	0	1	0	0	0	0	0	0	0	0
KLEBSIELLA PNEUMONIAE	0	1	2	4	3	7	2	4	0	0
KLEBSIELLA VARIICOLA	1	0	0	0	0	0	0	0	0	0
PSEUDOMONAS AERUGINOSA	0	1	4	2	4	1	0	1	0	0
SERRATIA MARCESCENS	0	1	0	0	0	0	0	0	0	0
STENOTROPHOMONAS MALTOPHILIA	1	3	0	1	0	0	0	0	0	0

MIC = minimum inhibitory concentration

(B)

Pathogen	Number of pathogen by MIC									
	MIC (µg/mL)									
	≤ 0.03	0.06	0.12	0.25	0.5	1	2	4	16	64
ACINETOBACTER BAUMANNII	1	0	3	3	6	2	2	1	0	1
ACINETOBACTER NOSOCOMIALIS	0	0	0	2	0	0	0	0	0	0
ACINETOBACTER PITTII	0	0	0	0	1	0	0	0	0	0
BURKHOLDERIA CEPACIA	1	0	0	0	0	0	0	0	0	0
CITROBACTER FREUNDII	0	0	0	0	1	0	0	0	0	0
CITROBACTER KOSERI	0	0	0	0	1	0	0	0	0	0
ENTEROBACTER AEROGENES	0	3	0	0	0	1	0	0	0	0
ENTEROBACTER ASBURIAE	0	0	1	0	0	0	1	0	0	0
ENTEROBACTER CLOACAE	0	0	0	0	2	0	1	0	0	0
ENTEROBACTER (non-speciated)	0	0	0	1	0	0	0	0	0	0
ESCHERICHIA COLI	5	4	2	1	4	1	0	0	0	0
HAEMOPHILUS INFLUENZAE	0	0	0	0	0	0	0	0	0	0
HAEMOPHILUS PARAHAEMOLYTICUS	0	0	0	0	0	0	0	0	0	0
HAFNIA ALVEI	0	0	0	0	0	1	0	0	0	0
KLEBSIELLA OXYTOCA	1	1	0	0	0	0	0	0	0	0
KLEBSIELLA PNEUMONIAE	7	3	0	3	8	7	6	2	0	0
MORAXELLA	0	0	0	0	0	0	0	0	0	0
MORAXELLA CATARRHALIS	0	0	0	1	0	0	0	0	0	0
MORGANELLA MORGANII	0	1	0	0	0	0	0	0	0	0
PROTEUS MIRABILIS	1	0	0	0	0	0	0	0	0	0
PSEUDOMONAS AERUGINOSA	1	3	7	5	3	1	0	0	0	0
RHIZOBIUM RADIOBACTER	0	0	0	0	0	0	0	0	1	0
SERRATIA MARCESCENS	1	0	0	4	1	0	0	0	0	0
STENOTROPHOMONAS MALTOPHILIA	0	0	0	1	0	0	0	0	0	0

MIC = minimum inhibitory concentration

Figure S1 Observed Plasma Concentration Profiles for Infected Patients by Study and Renal Function Group

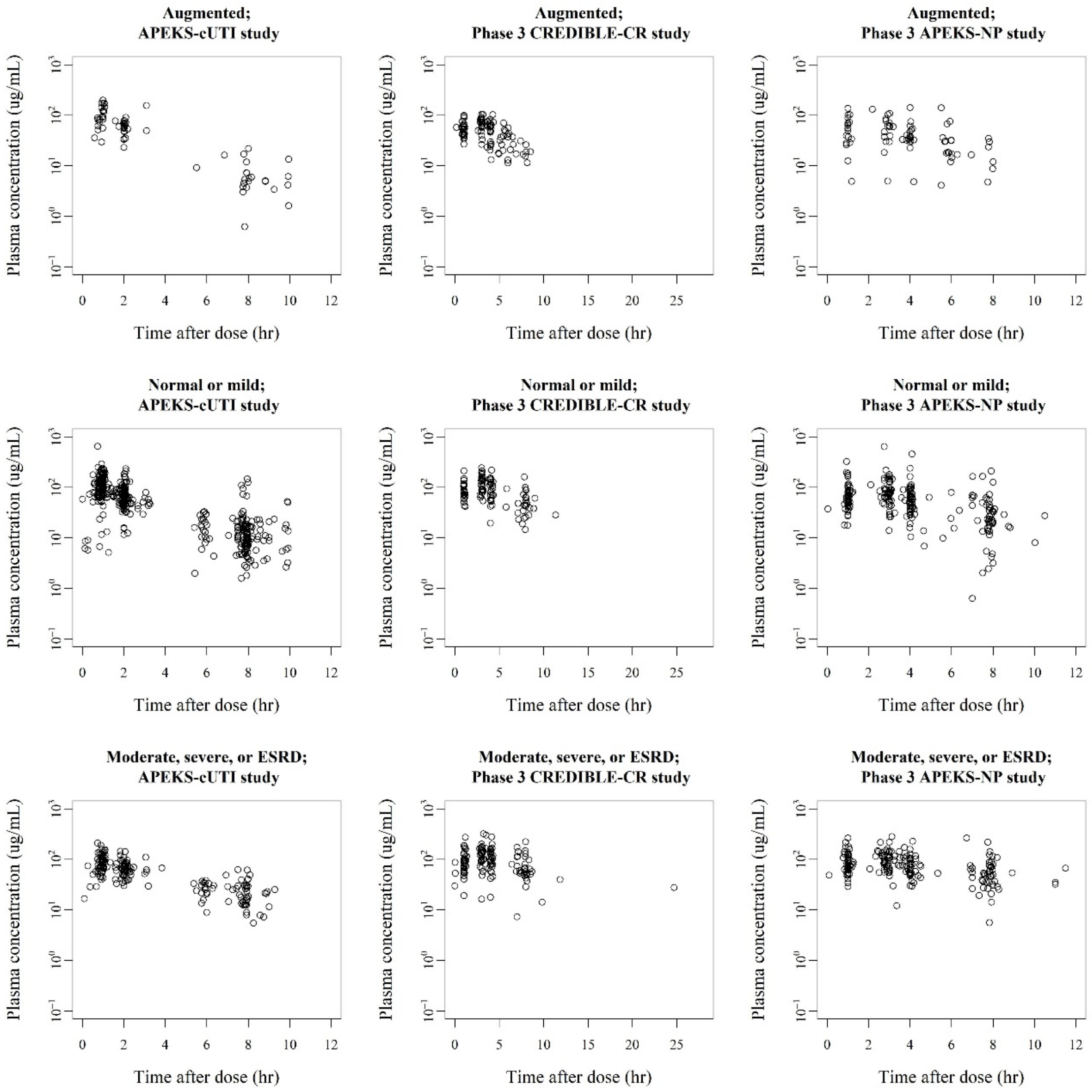

Figure S2 Goodness-of-fit Plots for Final Model

Solid line: y = x or y = 0. Red dashed line: a LOWESS line. Black dashed line: CWRESI = ± 6.

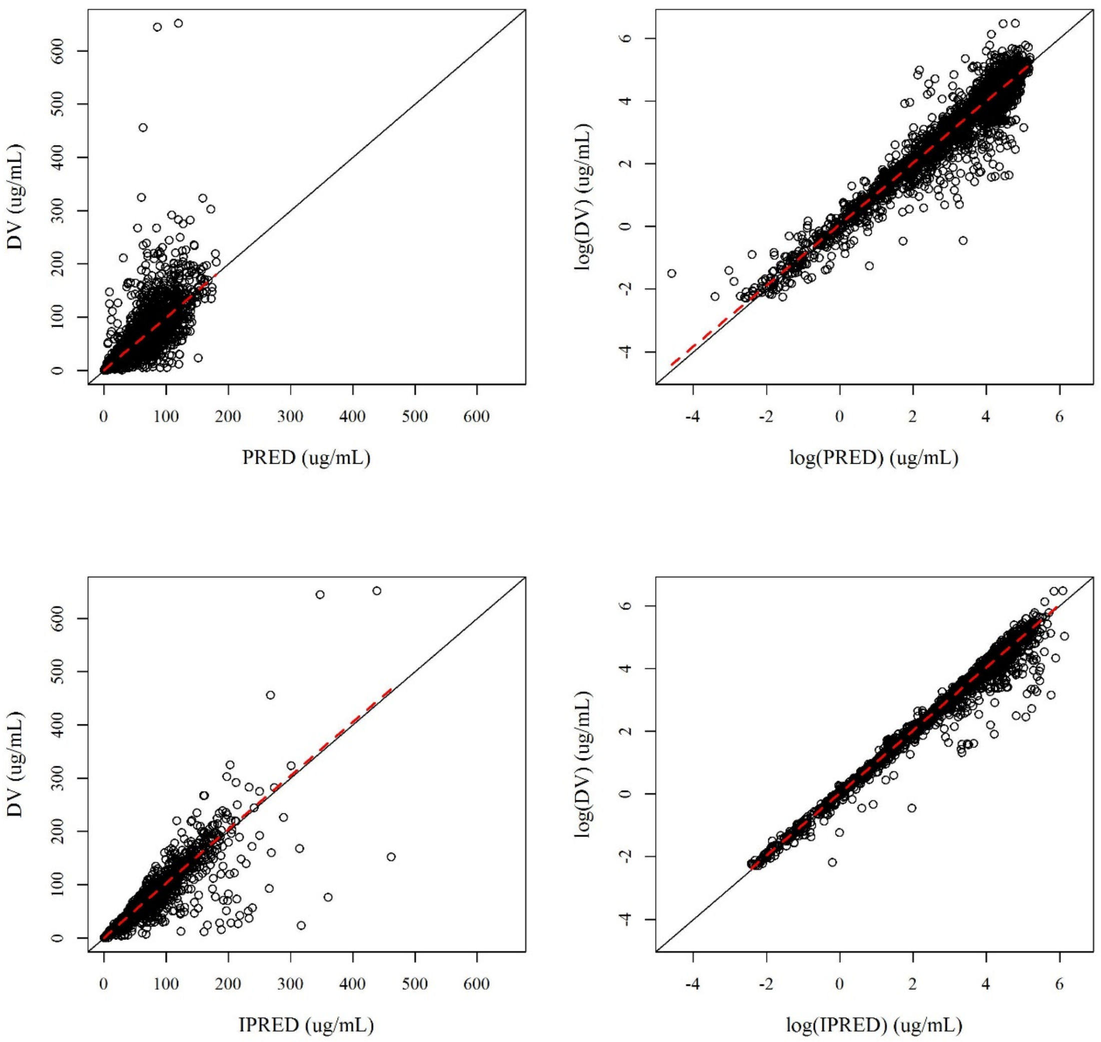

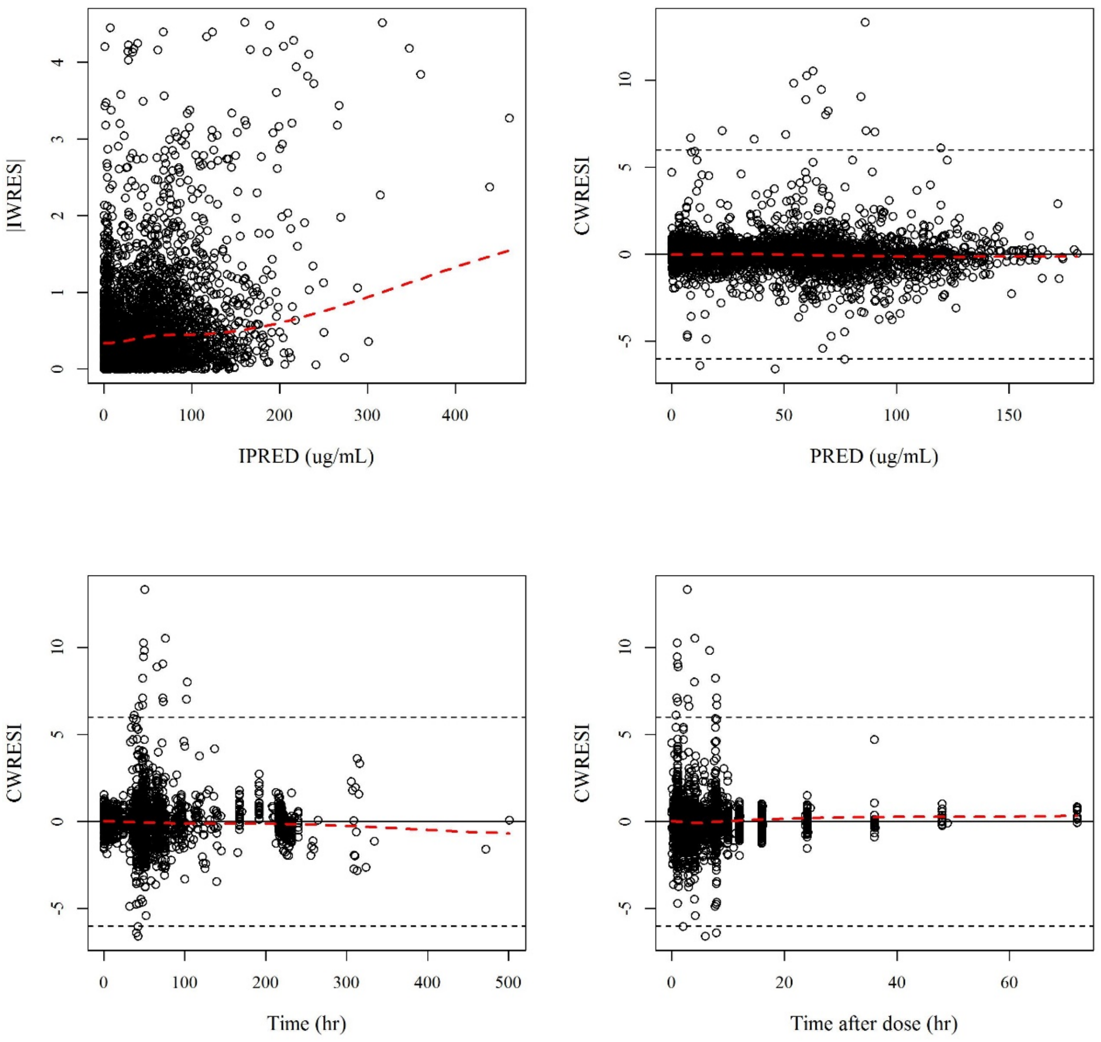

|IWRES|
IPRED (ug/mL)
CWRESI
PRED (ug/mL)
CWRESI
Time (hr)
CWRESI
Time after dose (hr)

Figure S3 Relationship of CL to CrCL for Base Model

Slid line: a LOWESS line. Dashed line: a correlation line.

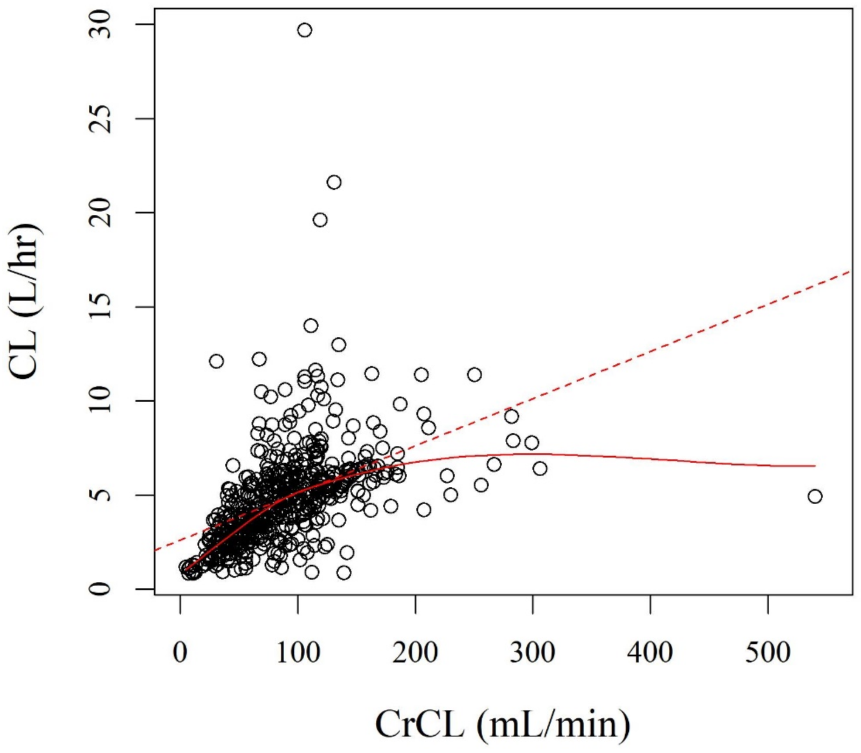

Figure S4 Box Plots for Estimated C_{max} and Daily AUC by Albumin Concentration Group for Patients in CREDIBLE-CR and APEKS-NP Studies

Red circle: post hoc estimates of parameters for individual patients. Horizontal black center line represents median, with the top and the base of the boxes representing first and third quartiles [interquartile range (IQR)], whiskers represent the most extreme data within 1.5 × IQR.

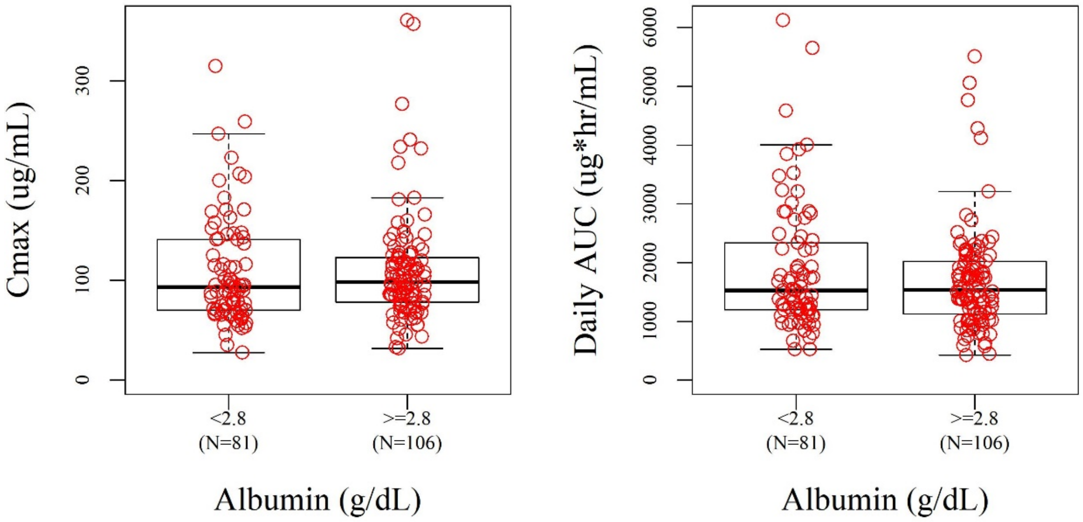

Figure S5 Box Plots for Estimated C_{max} and Daily AUC by Vital Status for Patients in CREDIBLE-CR and APEKS-NP Studies with Survival Cases in APEKS-cUTI Study

CR: CREDIBLE-CR study, NP: APEKS-NP study, cUTI: APEKS-cUTI study.
Red circle: post hoc estimates of parameters for individual patients. Horizontal black center line represents median, with the top and the base of the boxes representing first and third quartiles [interquartile range (IQR)], whiskers represent the most extreme data within 1.5 × IQR.

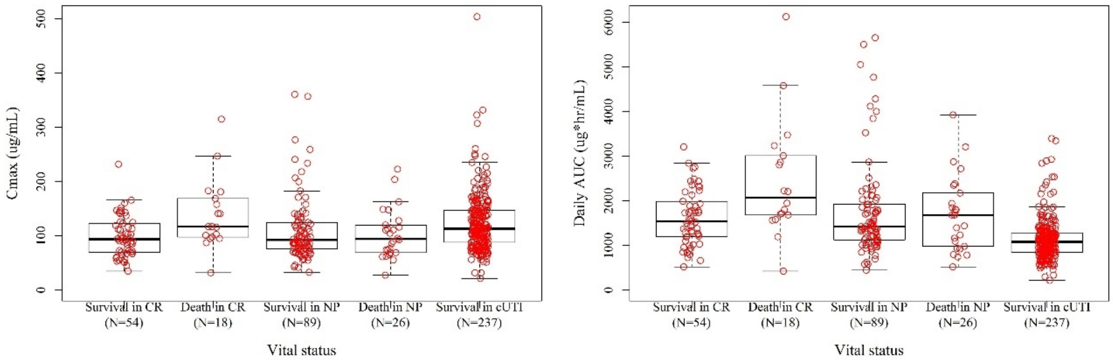

Supplement: Supplemental file 1 [file AAC.01437-20-s0001.pdf]
